# Hybrid Clustering and Routing Algorithm with Threshold-Based Data Collection for Heterogeneous Wireless Sensor Networks

**DOI:** 10.3390/s22155471

**Published:** 2022-07-22

**Authors:** Muhammad Bilal, Ehsan Ullah Munir, Fawaz Khaled Alarfaj

**Affiliations:** 1 Department of Computer Science, COMSATS University Islamabad, Wah Campus, Wah Cantt 47010, Pakistan; ehsan@ciitwah.edu.pk; 2 Department of Computer Science and Information Sciences, Imam Mohammad Ibn Saud Islamic University (IMSIU), Riyadh 11564, Saudi Arabia; fkarfaj@imamu.edu.sa

**Keywords:** centralized networks, distributed networks, threshold, network heterogeneity, deletion

## Abstract

The concept of the internet of things (IoT) motivates us to connect bulk isolated heterogeneous devices to automate report generation without human interaction. Energy-efficient routing algorithms help to prolong the network lifetime of these energy-restricted smart devices that are connected by means of wireless sensor networks (WSNs). Current vendor-level advancements enable algorithm-level flexibility to design protocols to concurrently collect multiple application data while enforcing the reduction of energy expenditure to gain commercial success in the industrial stage. In this paper, we propose a hybrid clustering and routing algorithm with threshold-based data collection for heterogeneous wireless sensor networks. In our proposed model, homogeneous and heterogeneous nodes are deployed within specific regions. To reduce unnecessary data transmission, threshold-based conditions are presented to prevent unnecessary transmission when minor or no change is observed in the simulated and real-world applications. We further extend our proposed multi-hop model to achieve more network stability in dense and larger network areas. Our proposed model shows enhancement in terms of load balancing and end-to-end delay as compared to the other threshold-based energy-efficient routing protocols, such as the threshold-sensitive stable election protocol (TSEP), threshold distributed energy-efficient clustering (TDEEC), low-energy adaptive clustering hierarchy (LEACH), and energy-efficient sensor network (TEEN).

## 1. Introduction

The modern era of electronics engineering, aviation, defense, robotics, computers, biomedical engineering, and meteorology enables researchers to design a variety of intelligent wireless sensors. The set of diminutive, low-cost, energy-efficient, self-organized, and programmable sensors are allied together to form a wireless sensor network (WSN). These networks are generally used to accumulate valuable information without human interaction in the domains of medical research, disaster management, engineering, tracking, industrial operations, and surveillance in battlefields [1]. Recent applications of WSNs introduced in the last decade include wave monitoring [2], ocean water monitoring [3], cattle herding [4], wildlife monitoring [5], landslide detection [6], and living and residential monitoring [7].

**Figure 1:**
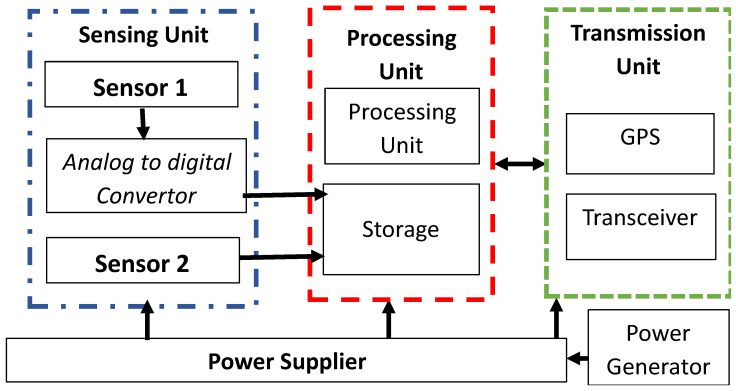
Layout of a node with multiple sensors.

One of the main limitations of WSNs is the limited power available at the sensor nodes because of their minuscule sizes, which cannot embed with massive capacity batteries, as shown in Figure 1. In most WSN applications, nodes are randomly deployed in the target environment, so it is impossible to collect and recharge them. Most of the prior literature on WSNs focuses on different techniques to conserve the available energy during computations and communications while collecting more valued data with stretched network stability.

**Figure 2:**
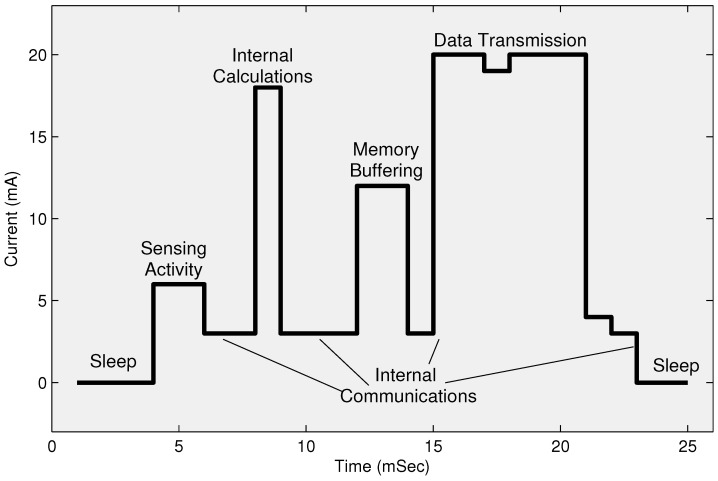
Average energy consumption in sensor node.

Figure 2 describes the phase-wise energy consumption of a sensor node. It shows that the communication phase’s energy dissipation is much higher than the energy consumption during internal computations and other internal operations.

**Figure 3:**
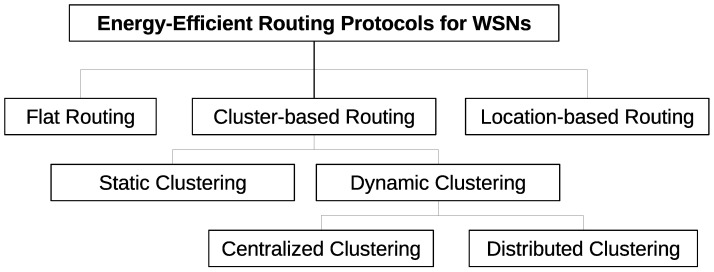
Classification of routing protocols in WSNs.

There are different energy-efficient routing techniques used in WSNs to gain enhanced performance. Each technique has its limitations, and the selection of appropriate topology depends upon the target application. Some common techniques are presented in Figure 3. The critical challenge of WSN is to adopt a technique that consumes the least energy and delivers accurate data collected from sensors to the sink node. Several algorithms have been proposed to reduce the computational complexities in data collection, aggregation, and delivery, and reduce the overall energy consumption of the sensor node [8].

The stability of WSNs depends upon the communication time period. That is only possible with efficient use of limited energy due to the architectural and communication restrictions of sensor nodes [9]. With the increase in the number of sensor nodes, and the coverage area of WSN, congestion in communication occurs. This congestion affects the delivery ratio of communication packets which can reduce their performance. Therefore, an efficient routing and congestion management are important to achieve effective performance.

Several energy-efficient data routing algorithms have been proposed in the past. Such algorithms are categorized into three classes: (a) flat routing [10], (b) location-based routing [11], and (c) hierarchical routing [12]. Further extensions with geographic routing algorithms have also been proposed to improve network stability [13,14,15,16].

The hierarchical clustering technique is more effective than the centralized or flat routing in WSNs. In this technique, the whole network is comprised of multiple clusters. A node is selected as the cluster head (CH) from each cluster using computational and probabilistic operations. The higher residual energy of a node results in higher chances of being selected as a cluster head. These CHs are responsible for collecting data from their respective clusters and transmitting the aggregated data to the BS. Some clustering schemes such as cluster formation, selection of CHs, and cluster size were always motivating the authors to research with new computational parameters. In static clustering, cluster formation is done only once that remains permanent until the entire network’s termination. Most of the routing algorithms focus on dynamic clustering in which clusters are re-created during the lifetime of WSN based on the current states of residual energy, node density, and other parameters.

**Figure 4:**
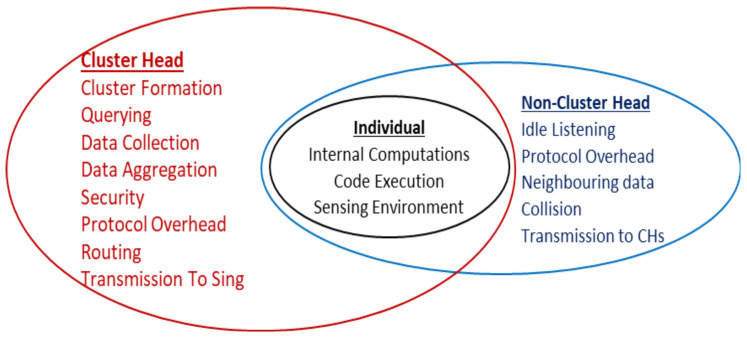
Common energy consuming tasks.

Each sensor node performs a few primary tasks, such as sensing environment, memory management, internal computing, and code execution. In WSNs, after cluster formation, there are different possible states of a node. A node can be (i) an independent node that is not a member of any cluster, (ii) a candidate for cluster head, (iii) a cluster head, (iv) a member node, (v) an assistant cluster head, (vi) a transient node that acts as a link between CH and cluster members, and (vii) a gateway node that acts as a linker between CH to CH. Additional responsibilities are assigned to the nodes in different states. Figure 4 describes the primary responsibilities of CHs, non-CHs, and individual sensor nodes. These additional tasks are also leading to a quick drain of the energy available at the sensor nodes. Since the transmission range of these miniature devices is very short, the placement of nodes and sink is one of the most challenging tasks in the formation of WSNs.

As most of a node’s energy is dissipated in the transmission phase as shown in Figure 2. If a CH is located far from the base station (BS) or sink, then it will drain more energy in the transmission phase than those CHs which are located closer to the BS. Therefore, several existing routing techniques have been extended to multi-hop routing techniques to manage coverage in larger areas and network stability. It is observed that the addition of nodes with higher energy levels exhibited enhanced performance. This heterogeneity of nodes in terms of residual energy provides more stability [17,18]. A variety of algorithms are discussed in the next section, including heterogeneous nodes. Some researchers focused on complex techniques for data aggregation to reduce the size of data transmission at CHs, which often overburdened the CH nodes and increased transmission delay.

In this paper, we focus on hierarchical routing in which an entire network is distributed in a group of nodes (clusters). A node is selected as the CH from each cluster using computational and probabilistic operations. These CHs are responsible for collecting data from their respective clusters and transmitting the aggregated data to the BS. By a few specified nodes, this controlled transmission shows significant improvement in the network stability by hierarchical (cluster-based) routing. We deploy homogeneous and heterogeneous nodes randomly within specific boundaries and collect multi-type information by clustering techniques. Furthermore, we use threshold values to regulate unnecessary transmission which shows more stability in our evaluation.

The rest of the paper is organized as follows. Section 2 describes related work and the background concepts relevant to our proposed technique. Section 3 describes the architecture of our proposed model and its phases. Section 4 provides the details of the proposed energy model. Section 5 presents the evaluation of the proposed approach. Finally, Section 6 concludes this paper.

## 2. Related Work

In the existing literature, several energy-efficient cluster-based routing protocols have been proposed to prolong the network lifetime. Low-energy adaptive clustering hierarchy (LEACH) [19], one of the pioneer cluster-based routing algorithms, focuses on the association of homogeneous nodes. Due to the random deployment of nodes and probabilistic operations to select CHs, some areas may contain closed CHs that lead to an unbalanced dense in a cluster. Thus the idea of dynamic clustering generates unintentional communication overhead. Similarly, the CHs that are located far from the BS require more energy to communicate. LEACH-centralized (LEACH-C) [20] unraveled this issue by selecting feasible CHs, by the BS, according to the network’s residual energy and the location of nodes. In this algorithm, the average residual energy of all alive nodes is first calculated. Those nodes are selected as CHs with higher residual energy than the calculated average network energy and are located at the most feasible positions within their respective clusters. The performance of LEACH-C exhibits the importance of the selection of cluster heads at feasible locations.

The threshold-sensitive energy efficient sensor network (TEEN) [21] introduces threshold-based techniques for communication to minimize the unnecessary transmission in energy-restricted networks. In TEEN, threshold parameters are selected by the sink node. Only those nodes are selected for communication, which can sense any change in the wireless network environment. Furthermore, most of the alive nodes are assumed dead when no change is reported for multiple consecutive rounds.

Most of the homogeneous networks die smoothly due to the same energy levels of wireless nodes [19,20,21]. Several efforts have demonstrated that the addition of a few different initial energy nodes (heterogeneous networks) can improve network stability. In [17], a few advanced energy nodes are added to the threshold-sensitive stable election protocol (TSEP) to prolong the network stability. High-level energy nodes are most likely to be selected as CHs; therefore, two different threshold parameters are used to select the CHs in TSEP due to two-level energy nodes. Later on, multi-level energy nodes with different residual energy levels are used in threshold distributed energy-efficient clustering (TDEEC) [18]. The CHs are selected according to their initial and residual energy levels with other probabilistic and threshold parameters, such as the average energy of the network in a particular round, average distance of CHs to the BS, and the average distance between cluster members to their respective CHs, within the TDEEC. Direct delivery of data packets from CH to BS, located at far distances, affects the performance of protocols in larger networks.

Resisting routing attacks and the trust security mechanism are applied in [22]. TAGA develops the node’s direct and indirect trust values considering neighboring nodes. Furthermore, researchers implemented a suitability function to choose the ideal cluster heads. The selection of CHs depends upon trust, which can change dynamically for secure routing.

In [23], the authors focused on the optimal location of CHs with gateway nodes to reduce energy consumption during the communication phase. After the selection of CHs, gateway nodes are selected using different parameters to find weightage. If the distance between CHs and BS is less than calculated thresholds, CHs directly forward packets to BS; otherwise, the gateway was responsible for delivering the packet to the next gateway or CHs. It requires extending the proposed model with multi-hopping to overcome the impact of scalability by selecting feasible intermediate nodes.

In [24], the authors introduced a genetic analysis approach. This approach utilizes a cluster node to choose the most favored sensor node at the most feasible position. Based on its efficient fitness capability measurements, it presented high accessibility and proficiency. The authors proposed efficient routing by returning collected information from cluster heads to cluster members with backbone information. An efficient selection of paths between nodes can enhance the network’s stability.

To enhance network stability, efficient use of routing paths is important. In order to select feasible paths, the authors suggest an energetically optimized multi-sink-based clustered WSN model combined with a fault-tolerant and energy-efficient model called the enhanced ACO-based routing protocol (EARP) [25]. This routing protocol addresses the various limitations of systems for detecting forest fires, including fault tolerance, network longevity, and response time.

The mobility of sink and network nodes highlighted more critical issues for researchers. In a recent article, a modified distance-predicated energy-cognizant (mDBEA) multi-hop routing protocol is suggested in [26]. This protocol is designed for mobile sinks and nodes and implemented in rivers with the concept of mobile ad hoc networks (MANET). It showed effective performance and could optimize network lifetime by reducing the energy consumption of the sensor nodes. In this strategy, the authors implemented dynamic adjustment of communication range between nodes to identify the most proximate path that utilizes the least amplitude of energy for transmission.

Several recent efforts [12,27] differentiate heterogeneous network environments with their functional limitations from the heterogeneity-based application parameters, such as multi-level energy, node connectivity, and computational node parameters, and describe their performance.

Network performance is differentiated by demonstrating single-hop or multi-hop topologies with energy-efficient CH selection techniques, application-specific heterogeneous nodes, and data security with reliable delivery to prolong network stability. Some efforts [28,29,30] focus on the node architecture and techniques to minimize energy depletion in different applications. Similarly, some researchers [31,32,33] explore the conceptual illustration to focus on multi-path routing techniques and their impact on packet duplication, throughput, and packet delivery to enhance network performance.

In [34], a hybrid technique of centralized and distributed clustering is proposed to enhance the stability of heterogeneous networks. It focuses on the deployment of homogeneous and multi-level energy nodes within two specific zones. The inner zone that contains homogeneous nodes follows the centralized clustering mechanism, and the area that contains heterogeneous nodes leverages distributed clustering techniques for CH selection and communication. This hybrid algorithm is focused on enhancing the energy-efficient data delivery and observing redundant packets from the same cluster in stable environments.

In [35], a load balancing technique is proposed as a straightforward routing algorithm with multi-level, multi-hop terminologies to reduce the energy consumption for data delivery at the base station. The authors divided the network environment into several circular fields to simplify the routing path by selecting the successive CHs in the network’s random and deterministic deployments. It requires dividing a larger network into several pieces for load balancing. An almost similar approach is implemented in [36,37]. CBCCP and its ME-CBCCP are clustering protocols with load balancing features to prolong lifetime. For larger areas, communication within the cluster is performed for the least energy consumption. The authors attempted a density-independent technique with the same cluster sizes that are applied with a multi-hop chaining tactic between cluster heads and cluster coordinating nodes to give packets at the BS. This sequencing of nodes offered enhanced outcomes with a larger number of connected IoT devices.

In [38], a 5G-based multiple-input multiple-output (MIMO) with multiple antennas is presented as a persuasive grouping approach for quickly developing IoT contexts to help a variety of client situations. It is a layering technique and each layer may contain multiple clusters. A single-hop and multi-hop communication were performed to cover larger zones effectively. In [39], a hybrid algorithm called whale optimization algorithm-moth flame optimization (MFO) is proposed to optimize the cluster formulation for IoT devices. MFO offers the advanced hybrid nature of CH selection, which is partially distributed and partially centralized to optimize the energy resources by saving the computational burden over network nodes. In our proposed model, some CHs use a centralized communication mechanism while others use a distributed communication scheme.

In [40], another hybrid routing algorithm is proposed for mobile IoT nodes with different velocities. The proposed model is derived from multipath optimized link state routing (OLSR) and ad hoc on-demand multipath distance vector (AOMDV) protocol design to develop the hybrid clustering algorithm.

After a review of the discussed articles [19,40], we observed that there are different parameters used to define the performance of energy-constrained wireless sensor networks. Some related issues that are focused on in our proposed model are:

- **Lifetime of Network:** The lifetime of a network can be measured in terms of the ratio between the available alive nodes and total nodes after specific periods. This is easy to measure, but special measures should be taken to improve its accuracy due to packet redundancy.
- **Span of Covered Area:** The performance of a WSN can be determined using the span of its covered area. However, this metric may not be workable for mission-critical environments because all nodes cannot successfully deliver accurate data to the sink or base station (BS) node, although nodes have sufficient energy to communicate with the neighboring nodes.
- **Connectivity Threshold:** A threshold can be used to measure connectivity or communication performance. This threshold is calculated using the parameters of alive nodes in terms of their residual energy, distance, and other communication factors. The threshold varies in different environments and applications.
- **Network Performance:** The performance of a WSN can be related to how long a network can receive data from the physical environment. Network performance can also be defined as the duration in which all nodes can communicate successfully with each other. This characteristic encourages heterogeneity in WSNs in terms of the capabilities of wireless nodes.

We observe in this section that most of the existing energy-efficient models are focused on data routing, data aggregation at CHs, and in successfully delivering the data packets to the BS. In our proposed model, we introduced a strategy to reduce packet transmission of cluster members to the CHs when the environment remains stable or changes gradually. We restrict member nodes of a cluster by using some conditions when communicating with CHs. This controlled transmission between member nodes and cluster heads will reduce the communication overhead of cluster heads and enhance the lifetime of the individual nodes in the network.

## 3. Proposed Model and its Phases

**Figure 5:**
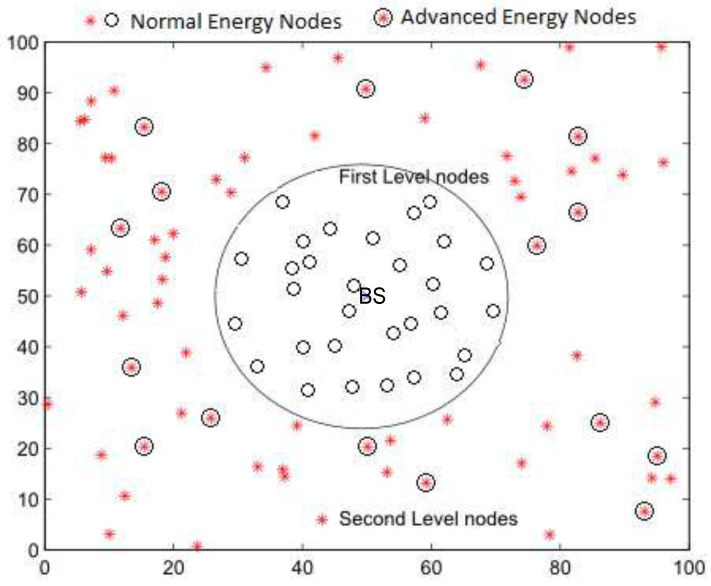
Energy-efficient routing protocols for WSNs.

In this section, we present the proposed network topology where the network is distributed between two physical levels called the *first level* and *second level*, as shown in Figure 5. In the first level, homogeneous nodes containing the same energy levels are deployed randomly within the base station’s target-oriented distance. At the time of network initialization, every node shares its location with BS to further realize its criteria of cluster formulation. It is mentioned in the flowchart that if the node is within the specified range, it will be marked as the first level, otherwise, it will be a second-level node. In the second level, located outside the inner zone, heterogeneous nodes in terms of initial normal and advanced energy levels are placed randomly. The BS node is located in the center of the network.

**Figure 6:**
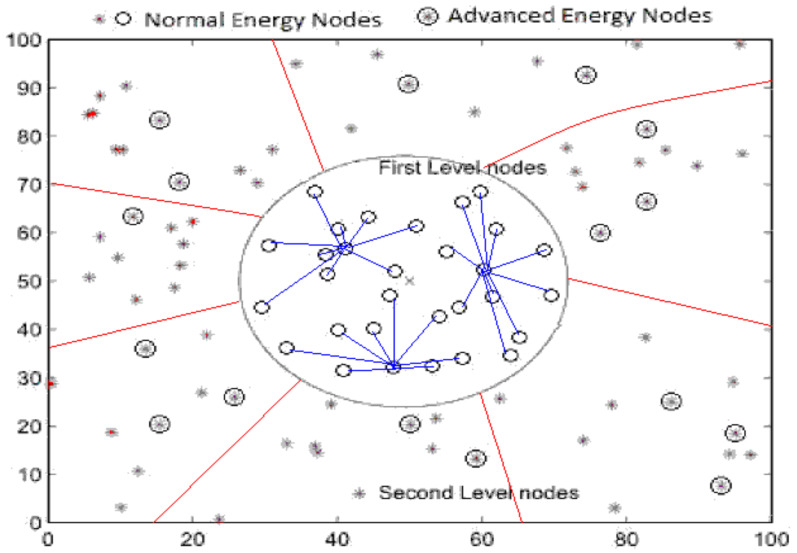
Clustering in energy-efficient routing protocols for WSNs.

The first-level region contains *m* homogeneous nodes deployed randomly within a specific range around the BS, and a centralized clustering technique is used to select the CHs. To improve network stability, *n* heterogeneous nodes with advanced initial energy levels are deployed in the second level, and a distributed cluster formation technique is applied in this region. Thus the whole network comprises *N* = m+n nodes. The clustering of nodes in different zones using this hybrid cluster formation technique is shown in Figure 6.

We have the following motivation for using this topology:

1. We placed BS in the center of WSN to assume that the center is often the most critical area of connectivity in the domains related to environmental, biological, and nuclear sciences.
2. Homogeneous nodes are easy to program, and their deployment is planned within a suitable area to conserve energy and to have easy access to the sink.
3. Heterogeneous nodes are a mixture of normal and advanced initial energy nodes. Thus, they are deployed far away from the BS. The advanced energy nodes have a higher probability of being selected as CHs, while the normal nodes will often be selected as the cluster members. This technique will help to improve network stability.

The proposed algorithm’s operating mechanism is divided into time-based slots (rounds), which are required for the cumulative network settling time and network transmission time in the cluster-based routing protocols. Each round contains multiple sub-phases called the cluster-head selection phase (CHSP), member association phase (MAP), and network transmission phase (NTP). An epoch is the execution time required to perform some specific algorithmic tasks in each round.

### 3.1. Cluster-Head Selection Phase (CHSP) in the First-Level Nodes

In the CHSP in the first level, *m* homogeneous nodes placed in a specific region near the BS and the network control are handed over to the BS, which plays a central role in selecting suitable cluster heads by the central property of the proposed hybrid algorithm. In this level, as BS keeps entire knowledge of the region, after computation the average energy in each round, BS compares the residual energy of each node to average energy and computes the weight of CHs selection in the central region. Meanwhile distributed area networks compute the probabilistic threshold to be selected as CH. In the first round, each node *i* has equal chances to select the CH due to equal initial energy levels. Each node will generate a random number from 0 or 1. If the generated number is less than $P_{\left(i\right)}$, the node will be selected as a CH.

(1) $$\begin{array}{c} P_{i}\left(t\right)=\left\{\begin{array}{cc} \frac{K}{m-K\times (r\times mod\frac{m}{K})}, & \mathrm{if}1\\ 0, & \mathrm{if}0 \end{array}\right. \end{array}$$

If the probability of selecting CH will be $P_{\left(i\right)}$ at time *t*, then the expectation of CH ($E_{ch}$) will help to compute the number of clusters as described in [41]:

(2) $$E_{ch}=\sum_{i=1}^{m}P_{i}\left(t\right)\times 1=K$$

The number of nodes that are not selected as CHs in the first round *r*, can be calculated by m−K×r. If *m* = 30 and *K* = 3, then non-CHs in round each round are 27, 24, 21, and so on. Due to the same residual energy, each node should be selected as CHs once on average after m/K rounds (10 rounds in the example) and these are not selected as CH in the recent rounds r×mod(m/K).

We consider $F_{i}\left(t\right)$ as an indicator function to represent either the node that can be selected as CH or a member node. If a node has $F_{i}\left(t\right)=1$, then the node is eligible to select as a CH; otherwise, not. Using this indicator function, the total number of nodes that can be selected as CHs at time *t* can be calculated as:

(3) $$E\left[\sum_{i=1}^{m}F_{i}\left(t\right)\right]=m-K\left(r\times mod\left(\frac{m}{K}\right)\right)$$

To select the most feasible cluster heads, we calculate the initial energy, residual energy, energy consumption ratio (ECR), and each node’s distance toward BS. If $E_{0}$ and $E_{r}$ represent the initial and residual energy in round *r*, for a centralized cluster formation, the energy consumption ratio (ECR) of the first level *m* nodes can be calculated as:

(4) $$ECR\left(m\right)=\frac{E_{0}}{E_{0}-E_{r}}$$

After calculating the ECR of each node, the distance from the BS ($d_{toBS}$) is calculated to determine the suitable eligible nodes selected as the CHs in the first level.

(5) $$Suitability\left(m\right)=\frac{E_{r}}{ECR\times d_{toBS}}$$

By substituting the value of Equation (4) in Equation (5):

(6) $$Suitability\left(m\right)=\frac{E_{r}}{\frac{E_{0}}{E_{0}-E_{r}}\times d_{toBS}}$$

Suppose *p* is the desired percentage of CHs in *m* nodes. In that case, the BS will select m×p CHs, which have more energy than the average residual energy of *m* nodes and have the highest suitability ratio within the first level of the network area.

### 3.2. Cluster-Head Selection Phase (CHSP) in the Second Level Nodes

At the second level, the distributed clustering technique is applied with *n* heterogeneous nodes simultaneously with the centralized cluster head section process in the first level *m* homogeneous nodes. The main reason to add heterogeneous nodes is to balance the network lifetime in both regions.

In the distributed scheme of cluster heads selection, at the time of network initialization, some preliminary information such as initial energy, residual energy, the nodes’ position, and the status of nodes within the whole network is exchanged between all nodes by broadcasting hello packets by gradual increasing the signal strength to detect neighboring nodes for cluster formation.

In the second level, two types of nodes are used concerning their residual energy. If *a* normal nodes have $E_{0}$ residual energy and *b* advanced nodes have α additional energy than the normal nodes, then each advanced node has $E_{0}\left(1+\alpha \right)$. At the start of the network, all advanced nodes are deployed with the same energy. Once the network starts collecting and broadcasting information, each node will consume its residual energy according to assigned processes. To find the average residual energy in the second level, we can calculate the total energy of normal and advanced nodes separately.

(7) $$E_{nor}=\sum_{i=1}^{a}E_{0}$$

(8) $$E_{adv}=\sum_{i=1}^{b}E_{0}\left(1+\alpha_{i}\right)$$

The total energy ($E_{total}$) in the second level can be find by Equations (7) and (8) as:

(9) $$E_{total}=E_{nor}+E_{adv}$$

where *i* is the rotating epoch in each time-based round. The average residual energy $\bar{E}\left(r\right)$ in an epoch $E_{i}$ can be calculated as:

(10) $$\bar{E}\left(r\right)=\frac{1}{n}\sum_{i=1}^{n}E_{i}$$

If $P_{opt}$ is the pre-determined number of CHs and $P_{i}$ is the probability of a node to be selected as a CH in the second-level nodes, then:

(11) $$P_{i}=P_{opt}\frac{E_{i}}{\bar{E}\left(r\right)}$$

The threshold *T* to select CHs can be calculated as:

(12) $$\begin{array}{c} T=\left\{\begin{array}{cc} \frac{P_{nor}}{1-P_{nor}\times \left(r\times mod\frac{1}{P_{nor}}\right)\times d}, & \mathrm{if}\;n\in G^{\prime }\\ \frac{P_{adv}}{1-P_{adv}\times \left(r\times mod\frac{1}{P_{adv}}\right)\times d}, & \mathrm{if}\;n\in G^{\prime \prime }\\ 0, & \mathrm{otherwise} \end{array}\right. \end{array}$$

where $G^{\prime }$ and $G^{\prime \prime }$ are the sets of eligible normal and advanced-energy nodes in a cluster which can be selected as CHs, *r* is the current round, and *d* is the distance from the BS, a random number is generated by each node $s_{i}$. If the value of $s_{i}$ is less than the threshold value in Equation (12), the node will be selected as CH; otherwise, the node will wait for the hello message from selecting CHs. As the second-level region comprises the heterogeneous nodes of two different residual energy levels, so the desired percentage value of the normal ($P_{nor}$) and advanced ($P_{adv}$) nodes can be calculated as:

(13) $$P_{nor}=\frac{P_{opt}}{1+\alpha m}$$

(14) $$P_{adv}=\frac{P_{opt}\left(1+\alpha \right)}{1+\alpha m}$$

In a similar fashion, the value of $P_{i}$ can be calculated for two-level heterogeneity as:

(15) $$\begin{array}{c} P_{i}=\left\{\begin{array}{cc} \frac{P_{opt}E_{I}\left(r\right)}{\left(1+\alpha m\right)\bar{E}\left(r\right)}, & \mathrm{if}\;\mathrm{node}\;\mathrm{is}\;\mathrm{normal}\\ \frac{P_{opt}\left(1+\alpha \right)E_{I}\left(r\right)}{\left(1+\alpha m\right)\bar{E}\left(r\right)}, & \mathrm{if}\;\mathrm{node}\;\mathrm{is}\;\mathrm{Advance} \end{array}\right. \end{array}$$

In multilevel heterogeneous networks, the formation of clusters is based on the energy of the nodes. The nodes at a higher level will have more energy than lower-level nodes and are likely to select as a CH. To manage this uncertainty, the probability of a single node in a multi-level heterogeneous network, $P_{i}$ can be calculated as:

(16) $$P_{i}=\frac{P_{opt}\times n\times \left(1+\alpha_{i}\right)}{n+\sum_{i=1}^{n}\alpha_{i}}$$

If *p* is the desired percentage of CHs, then the total number of cluster heads in the first-level and second-level regions can be calculated as:

(17) $$Total_{CHs}=\sum_{i=1}^{m\times p}CHs_{level_{1}}+\sum_{i=1}^{n\times p}CHs_{level_{2}}$$

### 3.3. Member Association Phase (MAP)

**Figure 7:**
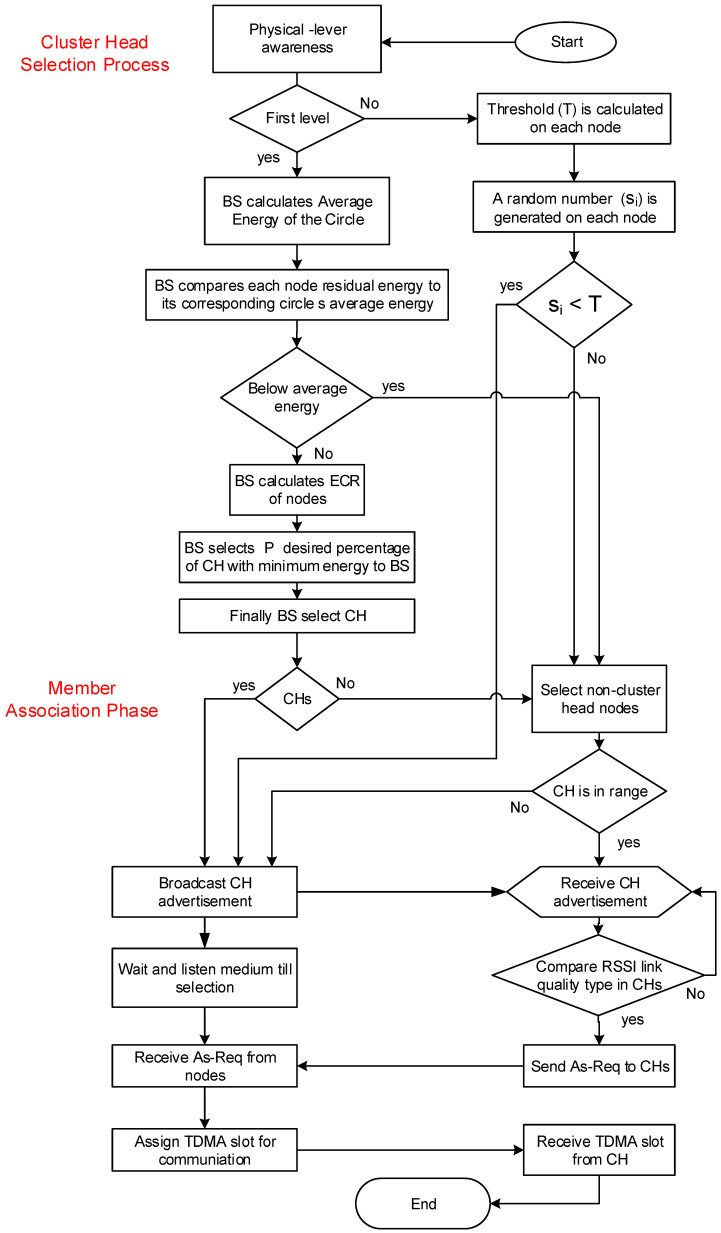
Flow control of the proposed cluster head selection and cluster formation techniques.

In the association phase, all selected cluster heads will broadcast hello messages to form clusters by utilizing the features of the carrier sense multiple access (CSMA) medium access control (MAC) protocol [42]. The other nodes remain active to respond to the transmitted hello messages. After receiving a hello message from the cluster head, a non-cluster head node will be selected as a cluster member node. It is also possible that a node receives multiple hello messages from multiple cluster heads. In this case, the signal strength is the node’s primary parameter to associate itself with the cluster head. i.e., a node associates itself with a cluster head with the most robust signal strength. The detailed MAP process is shown in Figure 7.

(18) $$Selec_{criteria}=\frac{RSSI}{Dis_{InRangeCHs}}$$

where RSSI is the received signal strength indication and $Dis_{InRangeCHs}$ is the distance between a non-cluster head node and the selected cluster head. Non-cluster heads select a cluster head with the maximum value of $Selec_{criteria}$ and send the association requests (As-Reqs) to the respective CHs using the CSMA MAC protocol. Simultaneously, cluster heads remain in a waiting state for association requests from non-cluster heads to form a cluster. After receiving all requests, the cluster head broadcasts a planned TDMA schedule to all member nodes. This TDMA schedule message from the nodes works as a confirmation report of their membership status in that cluster. These member nodes will only transmit their information when they reach their assigned periods. This period will be the active mode of the node. For the remaining duration, nodes deactivate their transmitter. This mode is called the sleep mode of the nodes.

### 3.4. Network Transmission Phase (NTP)

**Figure 8:**
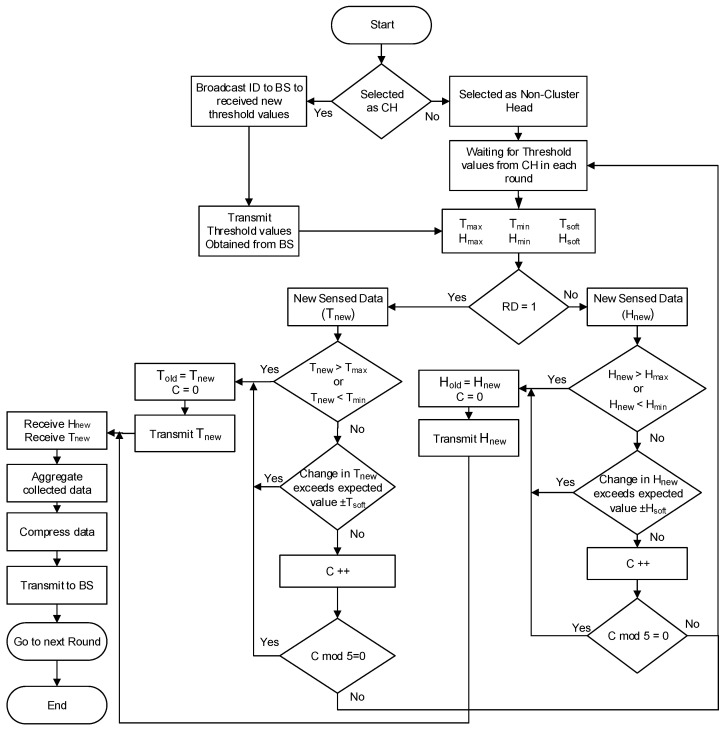
Flow control of the proposed network transmission phase.

In the network transmission phase (NTP), the TDMA schedule is created with cluster formation. Since the main objective here is to reduce the drain of node energy to prolong the transmission time of the network, we introduce a different mechanism by using threshold values in our algorithm to minimize energy dissipation when no or minor change is observed in the physical system. The proposed NTP process is shown in Figure 8.

In this paper, we consider two types of sensors available on the wireless nodes for collecting two types of data from the environment, temperature $T_{mp}$ and humidity $H_{mdy}$, which are distinguished by MAC addresses. The selection of a sensor for a node depends upon a boolean value RND in each round. If RND=1 then $T_{mp}$ will sense and broadcast updated value, if required, and if RND=0 then $H_{mdy}$ will broadcast updated sensed value, if required, to their respective cluster heads.

After forming clusters, each CH will broadcast parameters controlled by the BS in each round. In our proposed model, we use two types of threshold values adjusted by the BS in the NTP. The hard threshold represents the maximum ($T_{max}$, $H_{max}$) and the minimum ($T_{min}$, $H_{min}$) acceptable values of the network. The soft threshold represents small interval values ($T_{soft}$, $H_{soft}$) to prevent a node from communicating when minor or no change occurs. Furthermore, in our proposed model, when the system is stable, no transmission may be observed due to predefined threshold conditions. However, this may lead to an undesirable scenario when an alive node is considered a dead node [21]. To overcome this challenge, we introduce a counter (C) that is updated when a node performs no transmission for a fixed number of epochs. Afterward, the node will transmit its sense, and probably unchanged value, of the selected sensor to keep it alive for the WSN. The node resets the counter after each transmission.

If $T_{old}$ and $H_{old}$ are last transmitted values, $T_{new}$ and $H_{new}$ are sensed values in the current epoch, then the transmitting conditions for each sensor are shown in Figure 8. We define three possible conditions when a non-cluster head node will transmit updated information of the selected sensor to its CHs. These conditions are:

1. The hard threshold scenario when new sensed values of $T_{new}$ or $P_{new}$ are greater than the maximum accepting values ($T_{max}$, $H_{max}$) or lesser than the minimum accepting values ($T_{min}$, $H_{min}$).
2. The soft threshold scenario when the difference in previously transmitted values ($T_{old}$ and $H_{old}$) and new sensed values ($T_{new}$ or $P_{new}$) is unexpectedly larger or smaller than the expected change ($T_{soft}$, $H_{soft}$).
3. The stable network scenario when the network is stable, and the node is not transmitting its values ($T_{new}$ or $P_{new}$) since the last four consecutive numbers of rounds.

In the network’s initialization phase, the previously transmitted values of all nodes will be set as $T_{old}=0$ and $H_{old}=0$. The selected cluster heads will receive the values of hard threshold and soft threshold from the BS at CHSP and broadcast them to non-cluster heads during MAP.

In the first round, all nodes will initialize their values of one of the sensors, temperature or humidity, depending upon their self-generated RND value which is either 1 for the temperature sensor or 0 for the humidity sensor. Later on, the other sensor will be initialized at its turn.

The BS can change the threshold values after receiving a signal frame based on the predefined routines or using software-defined network (SDN) techniques [43].

After a few rounds, the network stability increases, which further reduces the inter-cluster communication resulting in a prolonged network lifetime.

Cluster heads aggregate the collected data from member nodes. As mentioned in Figure 2, most of the energy is consumed in data transmission which can overburden when the distance between CH and BS is longer. Thus, a variety of data compression techniques are used in WSNs and IoT. The CHs use a pre-configured application-oriented compression technique to reduce the size and quantity of data packets before transmission to BS. After compressing the aggregated data, as shown in Figure 8, cluster heads send this meaningful and updated information to the BS directly (single-hop) or indirectly via another intermediate node (multi-hop). The flow control of selecting a sensor, data sensing, and data transmission using our proposed model is shown in Figure 8.

### 3.5. Multi-Hoping Network Transmission

We extend our proposed model by introducing the multi-hoping technique during transmission and achieving network scalability. The multi-hop proposed model utilizes a distance-based intermediate CHs selection process and follows the threshold level of received signal strength indication (RSSI) of intermediate CHs. Initially, all CHs advertise their current location and RSSI information within a certain transmission range set by the network administrator. Remote CHs receive the following advertisements and compute the multi-hoping criteria (MHC). The MHC’s value is directly proportional to RSSI and inversely proportional to the mutual distance of intermediate CHs toward BS. The following equation computes the value of MHC:

(19) $$MHC=\frac{RSSI_{ICHs}}{Dis_{ICHs-BS}}$$

where $RSSI_{ICHs}$ is RSSI value intermediate CHs and $Dis_{ICHs-BS}$ is a mutual distance of intermediate CHs and BS. Along with multi-hoping assistance, the threshold-based actual data transmission of sensor nodes saves significant energy resources.

## 4. Energy Model of the Proposed Model

A sensor is a micro-electro-mechanical system (MEMS) [44] device that consumes its limited energy in sensing the environment, performing computations, and transmitting signals. The first-order energy model is used in the proposed model similar to the existing clustering protocols [17,18]. A considerable amount of energy is dissipated by the transmitter amplifier, indicated by $E_{amp}$ as shown in Figure 2. Let *d*, $d_{o}$, and *L* is destination distance, reference distance and packet size respectively, and $E_{TX}\left(L,d\right)$ is the total transmission energy consumption by a node. $E_{fs}d^{2}$ and $E_{amp}d^{4}$ represent free space and multipath models of wireless signals, respectively, and $E{}_{elec}$ represents the energy dissipation of transmitter circuitry. The total transmission energy can be calculated as:

(20) $$\begin{array}{c} E_{TX}\left(L,d\right)=\left\{\begin{array}{cc} L\times E_{elec}+L\times E_{fs}d^{2}, & \mathrm{if}d<d_{o}\\ L\times E_{elec}+L\times E_{amp}d^{4}, & \mathrm{if}d\geq d_{o} \end{array}\right. \end{array}$$

After each round (*r*), alive nodes have probability ($P_{i}$) to be selected as a CH. Therefore, the expected number of CHs ($E_{ch}$) can be calculated as:

(21) $$E_{ch}=\sum_{i=1}^{N}\left(P_{i}\left(r\right)\times 1\right)=K$$

where *N* is the total number of alive nodes and *K* is the number of clusters, the approximate number of nodes in each cluster is $N_{c}=N/K$. Therefore non-CHs ($n_{c}$) in each cluster will be $n_{c}=\left(N/K\right)-1$.

If $L_{c}$ is the data bits of one cluster, $E{}_{eleRX}$ is the energy consumption in receiving signals, and $d_{toCH}^{2}$ is the distance of nodes to their CHs, then the energy dissipated by non-CHs in a cluster can be calculated as:

(22) $$E_{nonCHs_{intra}}=\left(n_{c}\right)\left(E{}_{eleRX}\times L_{c}+E{}_{afs}\times L{}_{c}\times d_{toCH}^{2}\right)$$

If $mn_{c}$ is non-CHs that remain silent in a cluster under the described conditions, then $k_{c}$ = $n_{c}-mn_{c}$ will respond to CHs. The energy dissipated by $k_{c}$ nodes is:

(23) $$E_{nonCHs_{intra}}=\left(k_{c}\right)\left(E{}_{eleRX}\times L_{c}+E{}_{afs}\times L{}_{c}\times d_{toCH}^{2}\right)$$

The difference between Equation (22) and Equation (23) shows that fewer non-CH nodes may not respond to CHs, resulting in less energy dissipation in the data aggregation process. If all member nodes are responding to the CHs, then the energy dissipation in receiving and aggregating processes by a CH can be calculated as:

(24) $$\begin{array}{c} E_{CH_{intra}}=E{}_{eleRX}\times L_{c}+E_{data_{A}gg} \end{array}$$

The energy required to transmit the aggregated data from a CH to BS can be calculated as:

(25) $$\begin{array}{c} E_{CH_{inter}}=E{}_{eleTX}\times L{}_{c}+E{}_{afs}\times L{}_{c}\times d_{toBS}^{2} \end{array}$$

The total energy consumed by a cluster can be given as:

(26) $$E_{CT_{dissipation}}=E_{CH_{inter}}+E_{CH_{intra}}+E_{nonCHs_{intra}}$$

Finally, the overall network energy consumption by *K* clusters can be calculated as:

(27) $$E_{NT_{dissipation}}=K\times E_{CT_{dissipation}}$$

Equation (27) shows the maximum energy dissipation of a network in the first round during the network initialization. Later on, only those non-CHs will transmit their newly sensed data that justify the specified conditions, as shown in Figure 8. It is also possible that only CHs may transmit BS data when no change or minor changes occur in a cluster. In our evaluation, a variable number of transmitting nodes are observed in each cluster, depending on the user’s requirements and the frequency of the network’s physical changes. We reduce the energy consumption in two phases during the transmission phase in the first- and second-level nodes, first, by restricting non-cluster heads from transmitting duplicate packets to CHs as in Equation (23). Second, the CHs receive data from all or a subset of nodes in their clusters, which results in less energy consumption during computation and aggregation phases by each cluster head in the first-level and the second-level regions.

## 5. Performance Evaluation and Discussion

In this section, we evaluate the performance of our proposed clustering and routing models. We conduct our evaluation in a simulated environment with the following specifications. A total of N=100 nodes are randomly distributed in a 100 m × 100 m field. m=30 nodes are scattered in the first level physical circle with normal initial energy and range 30 m around the BS, and n=70 nodes place outside that circle. The range of the first level can adjust during the network initialization period depending upon the type of required information. The only condition of node deployment is that the first-level zone must contain the homogeneous nodes only. In the second level of the network, 20% nodes (b = 14 nodes) have advanced initial energy levels. The BS is located in the center of the network. During all communications, the first-order energy model is utilized, which is shown in Table 1. The nodes’ initial energy is set as 0.5 Joules for the homogeneous nodes of the first-level network region, and nodes in heterogeneous parts of the network have higher energy than the nodes in the first-level network. Each node consumes its limited energy during cluster formation, sensing, aggregation, and transmission or receiving data. Once the energy resources of a specific node reach zero, then it is automatically considered as dead. The initial energy values for the second-level network are selected randomly. The size of a packet is 4000 bits. The desired percentage of cluster heads remains the same in both of the network parts. The limit of cluster formulation of the proposed protocol is based on the desired percentage value. In this paper, we define the desired percentage at 10% of the total alive nodes. In a centralized region, this desired percentage is strictly forced by the BS. Meanwhile, in the distributed region, we use a probabilistic formula (Equation (16)) to compute the threshold for the nodes to be selected as CH. This probabilistic formula uses the value of the desired percentage (*p* = 0.1) to compute the threshold value. This threshold value is compared with the random number generated over the nodes participating in the clustering process. If the value of the random number of a node is less than the threshold value, then it is selected as CH. In this way we control the desired percentage of the CHs.

This paper collects two types of information: temperature and humidity, on the same node (programmable sensor DHT11) with hard threshold and soft threshold values. The selection of information depends upon the self-generated random number by each non-cluster head in each round. We first evaluate our proposed approach’s performance using the popular Network Simulator (NS) version 2.35 [45]. Our proposed model is tested by single-hop and multi-hop transmission techniques with the well-known existing clustering routing protocols, i.e., the hybrid advanced distributed and centralized clustering path planning algorithm (HADCC) [34], threshold distributed energy-efficient clustering (TDEEC) [18], threshold-sensitive stable election protocol (TSEP) [17], threshold-sensitive energy-efficient sensor network (TEEN) [21], low-energy adaptive clustering hierarchy (LEACH) [19], and LEACH-centralized (LEACH-C) [20].

We ignored the signal collision and interference effects with similar parameters for all protocols used in our evaluation to achieve more realistic and accurate results. The selected performance parameters are shown in Table 1.

For evaluation of a real-world scenario, we deployed a 100 m × 100 m wheat farm in the spring season (March 5–25) in the Potohar plateau of Pakistan. Table 2 shows the collected information in the real-world scenario. The performance of the proposed model shows almost similar trends as observed in simulations.

**Figure 9:**
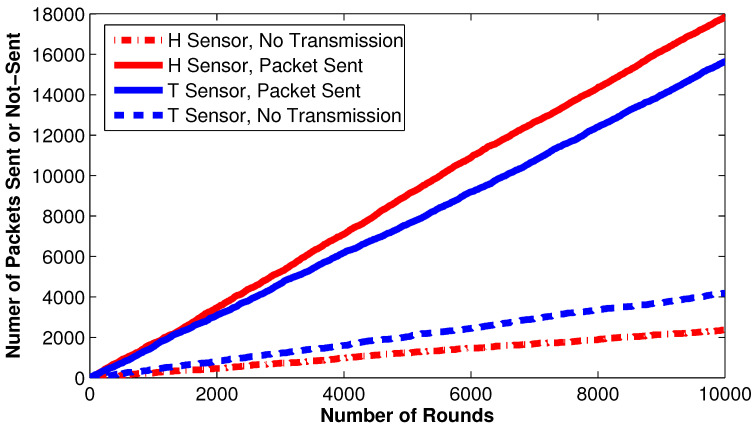
Number of packets sent by each sensor node with respect to the network operational rounds.

During the real-time experiment, a random sample of data is presented to explain the behavior of a cluster with a single hop is shared in Table 2. The number of packets sent by each sensor within a total number of rounds is shown in Figure 9. More than 20,000 attempts are observed when non-cluster heads perform no transmission due to the threshold conditions. The nodes’ behavior in a cluster containing eight alive non-cluster head nodes is shown in Table 2. The second, sixth, and seventh nodes broadcast values from the humidity sensor while other nodes transmit updated values from the temperature sensor. The first, fourth, sixth, and eighth nodes do not send updated information to the CH as similar information was sent in the recent rounds. However, these nodes update their counters. All other nodes broadcast the newly sensed values to the CH and reset their counters. The third node broadcast data due to the hard threshold condition that exceeds the accepting range of values. The remaining second, fifth, and seventh nodes transmit newly sensed values due to soft threshold conditions that demonstrate the system’s capabilities of managing quick or unpredictable changes in the environment.

### 5.1. Simulation Comparison of Single-Hop Proposed Model

**Figure 10:**
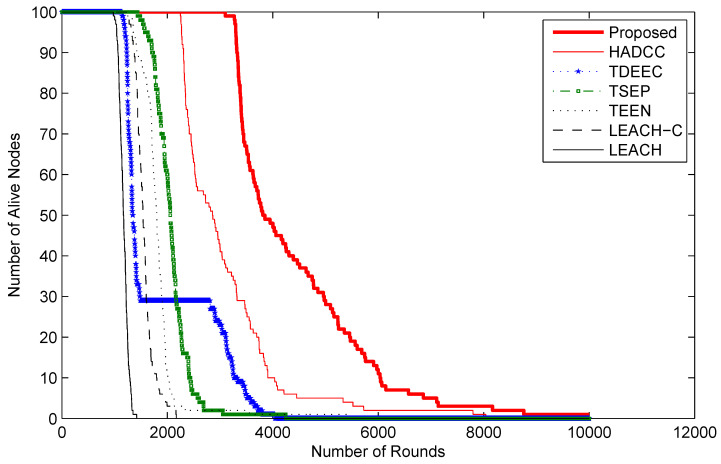
Number of alive nodes with respect to the network operational rounds.

Figure 10 shows the lifetime graph of nodes concerning the passing periods (rounds). The same node deployment policy is adopted in the experiment. We can observe that the proposed technique shows a longer stability period than LEACH, LEACH-C, TEEN, TSEP, TDEEC, and HADCC protocols. The number of alive nodes in LEACH, LEACH-C, and TEEN declined quickly because of homogeneous nodes. TSEP, TDEEC, and HADCC show more enhanced performance because of multi-level energy nodes. HADCC has similar network attributes but proposed restrictions to reduce transmission helped to prolong the network lifetime. Our proposed model does not allow the network nodes to transmit packets when the same or minor change is observed from the system. This strategy in our proposed algorithm assists to prolong life as compared to the other popular approaches.

**Figure 11:**
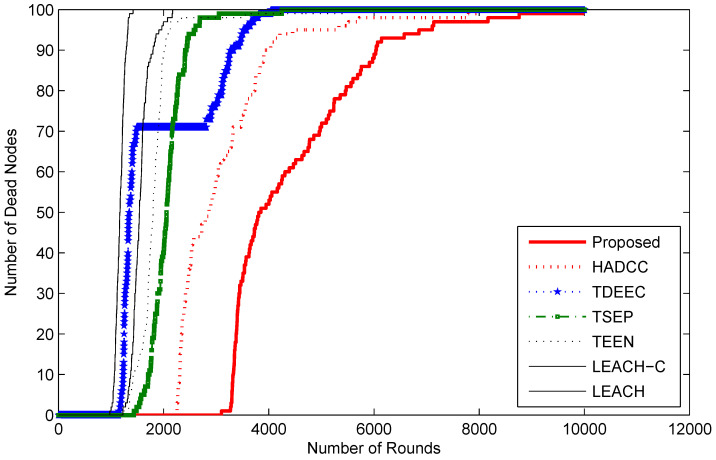
Number of dead nodes with respect to the network operational rounds.

Figure 11 shows the number of dead nodes as they run out of their limited energy. Our proposed model has more transmission time than LEACH, LEACH-C, TEEN, TSEP, and TDEEC protocols. The simulated results show that the networks with homogeneous nodes have low average energy and are wiped out too quickly. To prolong lifetime, nodes with advanced energy are used in TSEP, TDEEC, and HADCC. Smart deployment of nodes in HADCC and our scheme shows improved performance. As network topology of HADCC and our scheme is the same but threshold-based controlled communication helps to prolong network stability.

**Figure 12:**
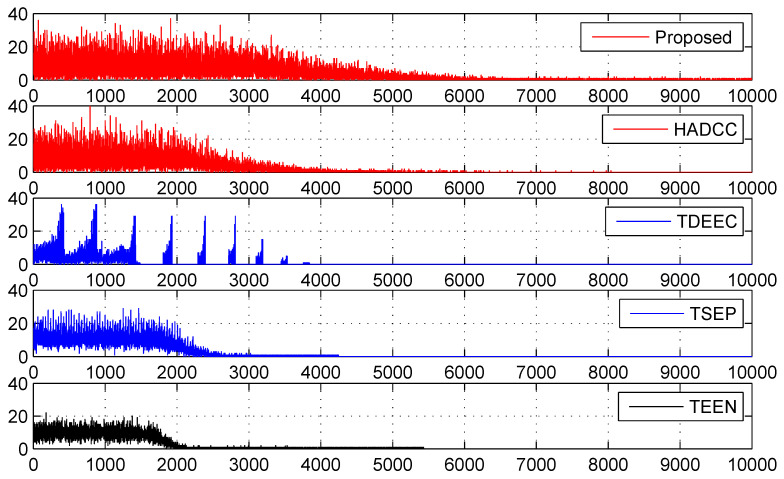
Number of cluster heads generated per round.

Next, we measure the number of cluster heads generated using various routing approaches that we use in our evaluation. In Figure 12, we ignored LEACH and LEACH-C to secure the space. The smallest energy drain helps to survive for a longer time. This stretched time period allows nodes to communicate and collect more information from the environment. We observe that our proposed algorithm shows enhanced performance because of the least consumption of restricted energy. This efficient use of energy leads to high network stability because of using the threshold technique for node placement in the WSN. This enhanced stability allows us to collect and communicate more information as compared to other protocols.

**Figure 13:**
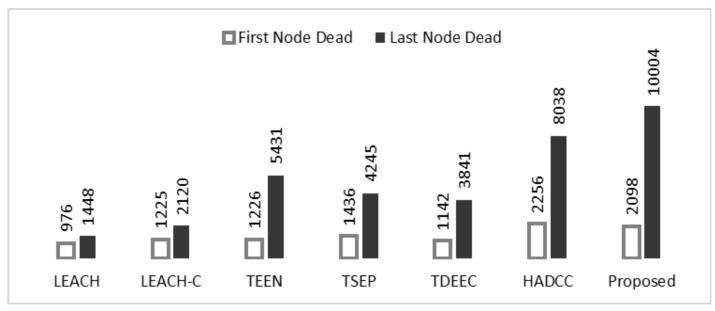
Performance evaluation of the proposed routing technique in terms of lifetime with other routing approaches.

Figure 13 shows the performance of network stability with the proposed technique and compares it with the other popular algorithms that we have used in our evaluation. Moreover, Table 3 shows a comparison of our proposed model with other well-known clustering and routing approaches for performance metrics critical in WSNs. The random deployment of nodes may require unnecessary broadcasting to detect CHs at a wide range. So few nodes are wasting energy like other schemes. The introduction of controlled communication enhanced the lifetime of our scheme. Overall, we observe that our proposed model significantly outperforms the other approaches used in our evaluation.

### 5.2. Simulation Comparison of Multi-Hop Proposed Model

**Figure 14:**
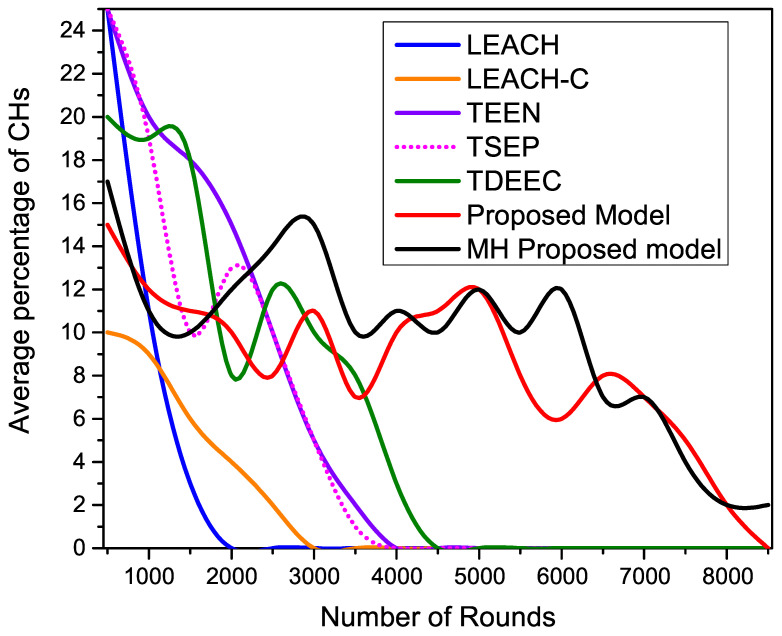
Average percentage of CHs generation during network operation.

Figure 14 shows the analytical analysis of the average percentage of CHs generated during the ground-based network operations. Our proposed and MH-proposed protocols show maximum stability in cluster formulation by maintaining the required percentage of CHs throughout the network operation. As compared to proposed protocols, all other existing distributed routing protocols show higher fluctuations of CHs generation. As network operation goes on, the number of alive nodes decreases rapidly, so all the protocols face clustering instability, but the proposed protocols successfully adjusted the desired percentage of the CHs.

**Figure 15:**
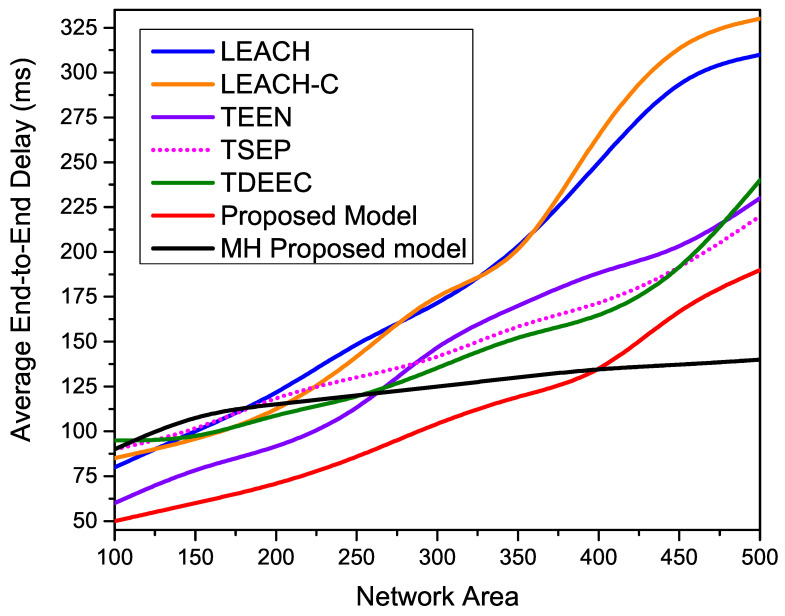
The average residual energy of CHs during network operation.

Figure 15 shows the residual energy of CHs during the network operation as we can observe that all the compared routing protocols have initial energy as residual energy at the start of the network. With the passage of network rounds, the residual energy starts decreasing. It reaches zero at the end of all the protocols’ network lifetime. The proposed protocols show maximum resistance and maintain even communication responsibilities among all the nodes to keep reasonable residual energy resources. The proposed protocols achieve maximum network lifetime due to better load-balancing among all the nodes.

**Figure 16:**
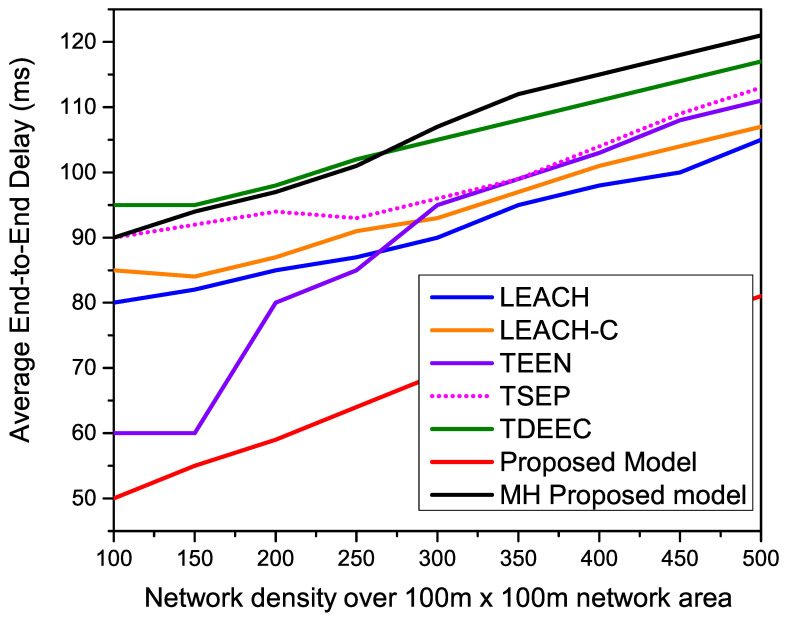
Performance evaluation of the average end-to-end delay for different network densities.

Figure 16 shows the average end-to-end delay of all compared routing protocols over different network area parameters. These simulation outcomes indicate the drastic performance degradation of all the single-hop routing protocols such as LEACH, LEACH-C, T-SEP, TEEN, and TDEEC once we increase the network. As compared to other single-hop routing protocols proposed model provides better results due to the hybrid nature of cluster formulation. The closer nodes follow centralized instructions; meanwhile, the out-circle nodes abide by the distributed property of CHs generating. The available CHs provide better management for collecting member nodes’ data and transmitting effectively to BS. However, the proposed MH-Proposed model provides more stable results as the network area increases.

**Figure 17:**
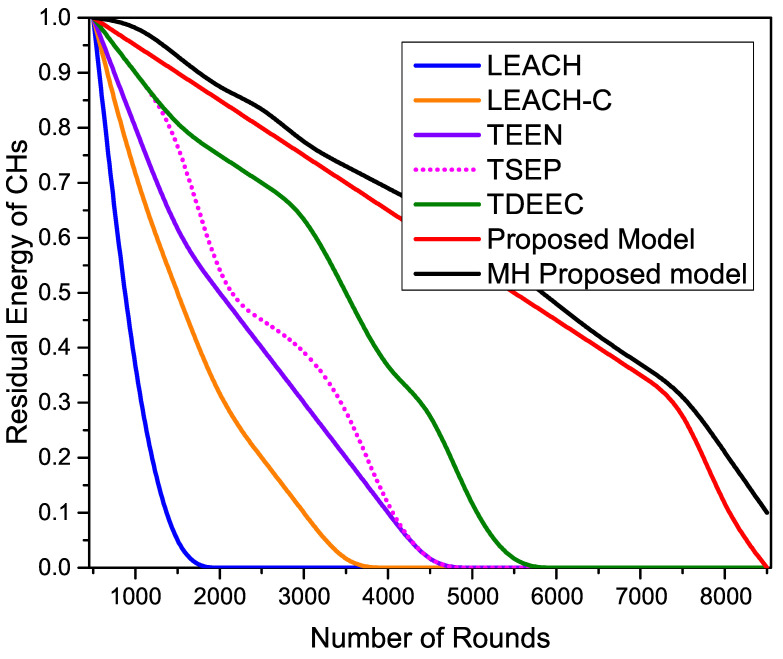
Performance evaluation of the average end-to-end delay for different network areas.

Figure 17 indicates the average end-to-end delay of all compared routing protocols over different network densities of 100, 150, 200, 250, 300, 350, 400, 450, and 500. Meanwhile, we keep the network area of 100 m × 100 m. All the single-hop routing protocols remain stable due to higher network density, resulting in a higher percentage of CHs. A higher percentage of CHs provides stable end-to-end delay during the network operation. Meanwhile, the proposed MH-proposed faces higher performance degradation due to the multi-hoping mechanism. This result indicates that multi-hopping is not suitable for smaller network areas.

## 6. Conclusions

In this paper, we presented a heterogeneity-aware, threshold-based hybrid clustering and routing algorithm that significantly improves the lifetime and the stability period of wireless sensor networks (WSNs) as compared to existing routing protocols, such as threshold-sensitive stable election protocol (TSEP), threshold distributed energy-efficient clustering (TDEEC), low-energy adaptive clustering hierarchy (LEACH), hybrid centralized clustering path planning algorithm (HADCC), LEACH-centralized and energy efficient sensor network (TEEN). Our proposed model contains a hybrid approach that provides a cluster head selection at two levels. Our hybrid clustering and routing algorithm adds flexibility in cluster formation based on the sensor node’s position, initial energy, residual energy, and cluster head selection history. These critical selection metrics for WSN provide a better cluster head selection and controlled network traffic. Moreover, our proposed technique addresses the challenges of centralized as well as distributed WSNs. Our proposed model utilized various thresholds, prevents redundant transmission of the same information, and improves the reliability of mission-critical WSNs.

The performance of the proposed model also depends upon the threshold values. It exhibits better results when the networks are stable as compared to the networks with frequent changes in their topology. Its performance depends upon the threshold values adjusted by the base station. We extended our proposal by introducing multi-hoping to achieve 10% to 20% better network scalability.

Although our proposed models have higher computational complexity as compared to the other distributed routing protocols, the performance evaluation shows that the proposed approach outperforms popular alternatives concerning the network lifetime, energy consumption, and overall network stability.

In our future work, we focus on its practical implementation for small and medium-sized farming areas to monitor the environment that often remains stable or gradual change is observed. We are focusing to collect the least information from member nodes to improve stability. We are expecting the enhanced performance of our proposed model that can acknowledge with the least communication traffic and time-critical features when unexpected or quick change occurs in the system.

## Figures and Tables

**Table 1:** Simulation parameters.

Parameter	Value
Network size	100 m × 100 m
BS position	50 m, 50 m
Total nodes *N*	100
First level nodes *m*	30
Second level nodes *n*	70
Normal nodes in *n* (80%)	56
Advanced nodes in *n* (20%)	14
*m* nodes/*n* nodes	30/70
Initial energy normal nodes	0.5 J
Initial energy advanced nodes	0.75 J
α	0.5
Desired percentage of CHs	0.1
Data aggregation energy cost	50 pJ/bit J
Packet size	4000 bit
$T_{hard}$/$T_{soft}$	20C–35C/2C
$H_{hard}$/$H_{soft}$	64%–80%/3%
EelecRx, EelectTx	50 nJ/bit
Transmit amplifier (Eamp)	100 pJ/bit/m^2^

**Table 2:** Temperature (T) and humidity (H) from 09:00 to 10:00.

Node	RND	Active Sensor	$T_{\mathrm{old}}$⇒$T_{\mathrm{new}}$	$H_{\mathrm{old}}$/$H_{\mathrm{new}}$	Counter	Transmit?
1	1	T	27 ⇒ 28	67 ⇒ -	2 ⇒ 3	No
2	0	H	23 ⇒ -	64 ⇒ 67	3 ⇒ 0	Yes
3	1	T	34 ⇒ 36	66 ⇒ -	2 ⇒ 0	Yes
4	1	T	30 ⇒ 31	68 ⇒ -	1 ⇒ 2	No
5	1	T	25 ⇒ 27	64 ⇒ -	2 ⇒ 0	Yes
6	0	H	30 ⇒ -	75 ⇒ 74	1 ⇒ 2	No
7	0	H	28 ⇒ -	68 ⇒ 70	1 ⇒ 0	Yes
8	1	T	29 ⇒ 30	70 ⇒ -	0 ⇒ 1	No

**Table 3:** Performance evaluation of the proposed technique.

Protocol	First Node Died	Last Node Died	Packets Sent
Proposed Model	3098	10,004	36,112
HADCC	2256	8039	24,092
TDEEC	1142	3841	32,815
TSEP	1436	4245	24,283
TEEN	1226	5431	12,283
LEACH-C	1225	2170	14,704
LEACH	976	1448	12,015

## Data Availability

The study did not report any data.

## References

[1] Asif M., Khan S., Ahmad R., Sohail M., Singh D. (2017). Quality of Service of Routing Protocols in Wireless Sensor Networks: A Review. IEEE Access.

[2] Marin-Perianu M., Chatterjea S., Marin-Perianu R., Bosch S., Dulman S., Kininmonth S., Havinga P. Wave monitoring with wireless sensor networks. Proceedings of the 2008 International Conference on Intelligent Sensors, Sensor Networks and Information Processing.

[3] Alkandari A., Alabduljader Y., Moein S.M. Water monitoring system using Wireless Sensor Network (WSN): Case study of Kuwait beaches. Proceedings of the 2012 Second International Conference on Digital Information Processing and Communications (ICDIPC).

[4] Kwong K.H., Wu T.T., Goh H.G., Sasloglou K., Stephen B., Glover I., Shen C., Du W., Michie C., Andonovic I. (2011). Implementation of herd management systems with wireless sensor networks. IET Wirel. Sens. Syst..

[5] Garcia-Sanchez A.J., Garcia-Sanchez F., Losilla F., Kulakowski P., Garcia-Haro J., Rodríguez A., López-Bao J.V., Palomares F. (2010). Wireless sensor network deployment for monitoring wildlife passages. Sensors.

[6] Kunnath A.T., Ramesh M.V. Integrating geophone network to real-time wireless sensor network system for landslide detection. Proceedings of the 2010 First International Conference on Sensor Device Technologies and Applications.

[7] Wood A.D., Stankovic J.A., Virone G., Selavo L., He Z., Cao Q., Doan T., Wu Y., Fang L., Stoleru R. (2008). Context-aware wireless sensor networks for assisted living and residential monitoring. IEEE Netw..

[8] Yetgin H., Cheung K.T.K., El-Hajjar M., Hanzo L.H. (2017). A survey of network lifetime maximization techniques in wireless sensor networks. IEEE Commun. Surv. Tutorials.

[9] Sabet M., Naji H.R. (2015). A decentralized energy efficient hierarchical cluster-based routing algorithm for wireless sensor networks. AEU-Int. J. Electron. Commun..

[10] Han G., Jiang J., Guizani M., Rodrigues J.J.C. (2016). Green routing protocols for wireless multimedia sensor networks. IEEE Wirel. Commun..

[11] Long J., Liu A., Dong M., Li Z. (2015). An energy-efficient and sink-location privacy enhanced scheme for WSNs through ring based routing. J. Parallel Distrib. Comput..

[12] Liu X. (2015). Atypical hierarchical routing protocols for wireless sensor networks: A review. IEEE Sensors J..

[13] Kang S.H. (2019). Energy optimization in cluster-based routing protocols for large-area wireless sensor networks. Symmetry.

[14] Gupta N.K., Yadav R.S., Nagaria R.K. (2020). 3D geographical routing protocols in wireless ad hoc and sensor networks: An overview. Wirel. Netw..

[15] Fanian F., Rafsanjani M.K. (2019). Cluster-based routing protocols in wireless sensor networks: A survey based on methodology. J. Netw. Comput. Appl..

[16] Vahabi S., Eslaminejad M., Dashti S.E. (2019). Correction to: Integration of geographic and hierarchical routing protocols for energy saving in wireless sensor networks with mobile sink. Wirel. Netw..

[17] Mittal N., Singh U., Sohi B.S. (2017). Harmony Search Algorithm Based Threshold-sensitive Energy-Efficient Clustering Protocols for WSNs. Adhoc Sens. Wirel. Netw..

[18] Rathore P., Kumar V. (2017). Optimization and Energy Efficient Analysis of Shortest Path Algorithm in WSN for Node Failure. Int. J. Comput. Appl..

[19] Zhai X.M., Yang L., Yang L. (2015). Research and Improvement of LEACH routing protocol. Applied Mechanics and Materials.

[20] Du T., Qu S., Liu F., Wang Q. (2015). An energy efficiency semi-static routing algorithm for WSNs based on HAC clustering method. Inf. Fusion.

[21] Abedin Z., Paul S., Akhter S., Siddiquee K.N.e.A., Hossain M.S., Andersson K. Selection of energy efficient routing protocol for irrigation enabled by wireless sensor network. Proceedings of the 2017 IEEE 42nd Conference on Local Computer Networks Workshops (LCN Workshops).

[22] Han Y., Hu H., Guo Y. (2022). Energy-Aware and Trust-Based Secure Routing Protocol for Wireless Sensor Networks Using Adaptive Genetic Algorithm. IEEE Access.

[23] Qureshi K.N., Bashir M.U., Lloret J., Leon A. (2020). Optimized Cluster-Based Dynamic Energy-Aware Routing Protocol for Wireless Sensor Networks in Agriculture Precision. J. Sensors.

[24] Rani S., Ahmed S.H., Rastogi R. (2020). Dynamic clustering approach based on wireless sensor networks genetic algorithm for IoT applications. Wirel. Netw..

[25] Moussa N., Benhaddou D., El Belrhiti El Alaoui A. (2022). EARP: An Enhanced ACO-Based Routing Protocol for Wireless Sensor Networks with Multiple Mobile Sinks. Int. J. Wirel. Inf. Netw..

[26] Abdulai J.D., Adu-Manu K.S., Katsriku F.A., Engmann F. (2022). A modified distance-based energy-aware (mDBEA) routing protocol in wireless sensor networks (WSNs). J. Ambient. Intell. Humaniz. Comput..

[27] Guleria K., Verma A.K. (2019). Comprehensive review for energy efficient hierarchical routing protocols on wireless sensor networks. Wirel. Netw..

[28] Engel A., Koch A., Siebel T. A heterogeneous system architecture for low-power wireless sensor nodes in compute-intensive distributed applications. Proceedings of the 2015 IEEE 40th Local Computer Networks Conference Workshops (LCN Workshops).

[29] Piromalis D., Arvanitis K. (2016). Sensotube: A scalable hardware design architecture for wireless sensors and actuators networks nodes in the agricultural domain. Sensors.

[30] Thoelen K. (2016). An Application Platform for Multi-Purpose Sensor Systems. https://limo.libis.be/primo-explore/fulldisplay?docid=LIRIAS1656578&context=L&vid=Lirias&search_scope=Lirias&tab=default_tab&fromSitemap=1.

[31] Laouid A., Dahmani A., Bounceur A., Euler R., Lalem F., Tari A. (2017). A distributed multi-path routing algorithm to balance energy consumption in wireless sensor networks. Ad Hoc Netw..

[32] Yim J., Bang J., Nam Y., Shin Y., Lee E. Efficient Multipath Routing Protocol Against Path Failures in Wireless Sensor Networks. Proceedings of the 2019 12th IFIP Wireless and Mobile Networking Conference (WMNC).

[33] Kim H., Shin Y., Lee Y., Shin D., Lee E. Event-to-Sink Multipath Routing Protocol for Event Reliability in Wireless Sensor Networks. Proceedings of the 2018 24th Asia-Pacific Conference on Communications (APCC).

[34] Aslam M., Munir E.U., Bilal M., Asad M., Ali A., Shah T., Bilal S. HADCC: Hybrid advanced distributed and centralized clustering path planning algorithm for WSNs. Proceedings of the 2014 IEEE 28th International Conference on Advanced Information Networking and Applications.

[35] Alaei M., Yazdanpanah F. (2019). EELCM: An Energy Efficient Load-Based Clustering Method for Wireless Mobile Sensor Networks. Mob. Netw. Appl..

[36] Rani S., Malhotra J., Talwar R. (2015). Energy efficient chain based cooperative routing protocol for WSN. Appl. Soft Comput..

[37] Rani S., Talwar R., Malhotra J., Ahmed S.H., Sarkar M., Song H. (2015). A Novel Scheme for an Energy Efficient Internet of Things Based on Wireless Sensor Networks. Sensors.

[38] Dogra R., Rani S., Babbar H., Krah D. (2022). Energy-Efficient Routing Protocol for Next-Generation Application in the Internet of Things and Wireless Sensor Networks. Wirel. Commun. Mob. Comput..

[39] Maddikunta P.K.R., Gadekallu T.R., Kaluri R., Srivastava G., Parizi R.M., Khan M.S. (2020). Green communication in IoT networks using a hybrid optimization algorithm. Comput. Commun..

[40] Deebak B.D., Al-Turjman F. (2020). A hybrid secure routing and monitoring mechanism in IoT-based wireless sensor networks. Ad Hoc Netw..

[41] Yang M., He J., Zhang Y. (2014). Calculating the Number of Cluster Heads Based on the Rate-Distortion Function in Wireless Sensor Networks. Sci. World J..

[42] (1987). IEEE Standards for Local Area Networks: Supplements to Carrier Sense Multiple Access with Collision Detection (CSMA/CD) Access Method and Physical Layer Specifications.

[43] Kalkan K. (2020). SUTSEC: SDN Utilized Trust based Secure Clustering in IoT. Comput. Netw..

[44] Hamami L., Nassereddine B. A Study of the Main Factors Affecting Wireless Sensor Networks. Proceedings of the 2019 Third International conference on I-SMAC (IoT in Social, Mobile, Analytics and Cloud)(I-SMAC).

[45] Issariyakul T., Hossain E. (2010). Introduction to Network Simulator NS2.

